# AnimalTFDB 4.0: a comprehensive animal transcription factor database updated with variation and expression annotations

**DOI:** 10.1093/nar/gkac907

**Published:** 2022-10-21

**Authors:** Wen-Kang Shen, Si-Yi Chen, Zi-Quan Gan, Yu-Zhu Zhang, Tao Yue, Miao-Miao Chen, Yu Xue, Hui Hu, An-Yuan Guo

**Affiliations:** Center for Artificial Intelligence Biology, Hubei Bioinformatics & Molecular Imaging Key Laboratory, Key Laboratory of Molecular Biophysics of the Ministry of Education, College of Life Science and Technology, Huazhong University of Science and Technology, Wuhan, Hubei 430074, China; Center for Artificial Intelligence Biology, Hubei Bioinformatics & Molecular Imaging Key Laboratory, Key Laboratory of Molecular Biophysics of the Ministry of Education, College of Life Science and Technology, Huazhong University of Science and Technology, Wuhan, Hubei 430074, China; Institute of Hematology, Union Hospital, Tongji Medical College, Huazhong University of Science and Technology, Wuhan, Hubei 430074, China; Center for Artificial Intelligence Biology, Hubei Bioinformatics & Molecular Imaging Key Laboratory, Key Laboratory of Molecular Biophysics of the Ministry of Education, College of Life Science and Technology, Huazhong University of Science and Technology, Wuhan, Hubei 430074, China; Center for Artificial Intelligence Biology, Hubei Bioinformatics & Molecular Imaging Key Laboratory, Key Laboratory of Molecular Biophysics of the Ministry of Education, College of Life Science and Technology, Huazhong University of Science and Technology, Wuhan, Hubei 430074, China; Center for Artificial Intelligence Biology, Hubei Bioinformatics & Molecular Imaging Key Laboratory, Key Laboratory of Molecular Biophysics of the Ministry of Education, College of Life Science and Technology, Huazhong University of Science and Technology, Wuhan, Hubei 430074, China; Center for Artificial Intelligence Biology, Hubei Bioinformatics & Molecular Imaging Key Laboratory, Key Laboratory of Molecular Biophysics of the Ministry of Education, College of Life Science and Technology, Huazhong University of Science and Technology, Wuhan, Hubei 430074, China; Center for Artificial Intelligence Biology, Hubei Bioinformatics & Molecular Imaging Key Laboratory, Key Laboratory of Molecular Biophysics of the Ministry of Education, College of Life Science and Technology, Huazhong University of Science and Technology, Wuhan, Hubei 430074, China; Center for Artificial Intelligence Biology, Hubei Bioinformatics & Molecular Imaging Key Laboratory, Key Laboratory of Molecular Biophysics of the Ministry of Education, College of Life Science and Technology, Huazhong University of Science and Technology, Wuhan, Hubei 430074, China; Department of Laboratory Medicine, Tongji Hospital, Tongji Medical College, Huazhong University of Science and Technology, Wuhan, Hubei 430074, China; Center for Artificial Intelligence Biology, Hubei Bioinformatics & Molecular Imaging Key Laboratory, Key Laboratory of Molecular Biophysics of the Ministry of Education, College of Life Science and Technology, Huazhong University of Science and Technology, Wuhan, Hubei 430074, China; Department of Laboratory Medicine, Tongji Hospital, Tongji Medical College, Huazhong University of Science and Technology, Wuhan, Hubei 430074, China

## Abstract

Transcription factors (TFs) are proteins that interact with specific DNA sequences to regulate gene expression and play crucial roles in all kinds of biological processes. To keep up with new data and provide a more comprehensive resource for TF research, we updated the Animal Transcription Factor Database (AnimalTFDB) to version 4.0 (http://bioinfo.life.hust.edu.cn/AnimalTFDB4/) with up-to-date data and functions. We refined the TF family rules and prediction pipeline to predict TFs in genome-wide protein sequences from Ensembl. As a result, we predicted 274 633 TF genes and 150 726 transcription cofactor genes in AnimalTFDB 4.0 in 183 animal genomes, which are 86 more species than AnimalTFDB 3.0. Besides double data volume, we also added the following new annotations and functions to the database: (i) variations (including mutations) on TF genes in various human cancers and other diseases; (ii) predicted post-translational modification sites (including phosphorylation, acetylation, methylation and ubiquitination sites) on TFs in 8 species; (iii) TF regulation in autophagy; (iv) comprehensive TF expression annotation for 38 species; (v) exact and batch search functions allow users to search AnimalTFDB flexibly. AnimalTFDB 4.0 is a useful resource for studying TF and transcription regulation, which contains comprehensive annotation and classification of TFs and transcription cofactors.

## INTRODUCTION

Transcription factors (TFs) are proteins with DNA-binding domains (DBDs) that recognize specific DNA sequences to regulate gene expression and affect almost all biological processes (1). Accurate identification and comprehensive annotation for TFs are key prerequisites and basis for studying TF functions and gene expression regulation. In response to the demand of systematical identification and annotation of TFs, several dedicated TF databases have been developed. For example, PlantTFDB (2,3) is the most comprehensive plant TF database, which has identified and well-annotated TFs for 165 plant species. For animal TF databases, The Human Transcription Factors database (1) and REGULATOR (4) contain TF information for a single genome and 82 metazoan species, respectively. There are also some disease-related TFs databases, including DBTFLC (5) and BC-TFdb (6), that identified TFs associated with lung cancer and breast cancer, respectively. Among all the TF databases, Animal Transcription Factor Database (AnimalTFDB) is the most comprehensive animal TF database including classification and annotation of genome-wide TFs and transcription cofactors (TcoFs). We constructed the AnimalTFDB in 2011 (7), and updated it in 2015 (8) and 2019 (9) with more species, annotations and functions. AnimalTFDB has become an essential resource for studying animal TFs and regulations, accessed by millions and cited over 650 times.

As one of the essential regulator types in various biological processes, TFs have been studied in many areas, including gene expression regulation (10), conservation or evolution (11), genetics or diseases (12), TF regulatory networks (13) and TF target prediction (14). Recently, the functions of TF in autophagy, post-translational modifications (PTMs) and variations in diseases have been determined, enhancing our understanding of the biological process influenced by TFs. Variations associated with TFs could frequently result in impaired transcriptional activation of TFs (15). ClinVar (16) and COSMIC (17) databases identified and integrated human variations in cancers and other diseases, which are vital resources for studying TF-related variations. In the past four years, the number of species in Ensembl (18) database has approximately doubled. Thus, we upgraded AnimalTFDB to version 4.0 with the latest genomes, annotation data and new functions. Compared with previous versions, AnimalTFDB 4.0 covers more species, more TFs and TcoFs with updated annotation data. In addition, we not only integrated the TF-related variations (mutations), gene expression, PTMs and autophagy information, but also provided the exact and batch search functions. The new AnimalTFDB 4.0 will be a helpful resource for transcriptional regulation and comparative genomics research.

## DATA SOURCE

In AnimalTFDB 4.0, we downloaded all the protein sequences of 183 animal genomes from Ensembl (version 105). These 183 species are classified into 12 catalogs according to their taxonomy, namely ‘Afrotheria’, ‘Amphibians’, ‘Birds&Reptiles’, ‘Fishes’, ‘Laurasiatheria’, ‘Other Chordates’, ‘Other Eukaryotes’, ‘Other Mammals’, ‘Other Vertebrates’, ‘Primates’, ‘Rodents’ and ‘Xenarthra’ (Supplementary Table S1). Compared with AnimalTFDB 3.0, catalogs with a large increase in the number of species are ‘Fishes’ (from 11 to 59 species) and ‘Birds&Reptiles’ (from 7 to 24 species).

We collected a large quantity of annotations from the NCBI Entrez Gene (19) and Ensembl (18) databases, including basic gene information, homologous genes, gene phenotypes and Gene Ontology (GO). We acquired genome-wide association studies (GWAS) phenotypes from the latest GWAS Catalog (20) and dbSNP (release 155) (19). Protein-protein interaction (PPI) data were gathered from BioGRID (version 4.4) (21) and HPRD (22), and the protein functional domains were predicted by PfamScan for all protein domain models in the Pfam (version 35.0) database (23). We obtained the signaling pathway information from BioCarta and KEGG (24) databases. Furthermore, transcription factor binding site (TFBS) information was extracted from HOCOMOCO (25), TRANSFAC (26), JASPAR (27) and CIS-BP (28) databases.

Next, we integrated 190 627 and 8 294 851 variation records for TFs and TcoFs from ClinVar (16) and COSMIC (v96) (17), respectively. PTM information of TFs and TcoFs for eight species was obtained from CPLM (29) and EPSD (30), containing 131 378 phosphorylation sites and 38 943 lysine modification sites (including acetylation, methylation and ubiquitination). In addition, we accessed information from THANATOS (31) on whether a TF or TcoF is involved in regulating autophagy-related processes (autophagy, apoptosis, and necrosis). Moreover, TF expression data for 38 species were collected from TCGA (32), EMBL-EBI Expression Atlas (33), GTEx (34), Bgee (Version 15.0) (35), FANTOM5 (36), the Human Protein Map (37), the Human Protein Atlas (38) and some articles with large-scale expression data (39–41). In AnimalTFDB 4.0, the number and types of data are more comprehensive than in previous version (Table 1).

**Table 1.** The comparisons on data volume, annotation and tool between version 3.0 and 4.0 of AnimalTFDB database

AnimalTFDB		Version 3.0	Version 4.0
**Data volume**	Species	97	183
	TF families	73	73
	TF genes	125 135	274 633
	Cofactor genes	80 060	150 726
	Cofactor families	83	82
**Annotations**	Species with expression data	22	38
	Phenotype	Yes	Yes
	DBDs WebLogo	Yes	Yes
	TFBS	Yes	Yes
	GWAS	Yes	Yes
	Variations	No	Yes
	Autophagy	No	Yes
	PTM	No	Yes
**Tools**	TF prediction server	Yes	Yes
	BLAST search	Yes	Yes
	PPI network	Yes	Yes
	TFBS prediction server	Yes	Yes
	Exact and batch search	No	Yes

## IMPROVED TF/COFACTOR FAMILY RULES AND PREDICTION PIPELINE

### The classification and assignment rules for animal TF families

TFs are usually classified into different families according to their conserved DBDs. In AnimalTFDB 4.0, we classified TFs into 73 families and six categories, which are consistent with AnimalTFDB 3.0. We optimized the TFs family assignment rules in AnimalTFDB 3.0 by manually checking the results in human and mouse genomes. We made several rules to classify a TF into its correct family. First, we classified the TFs based on the family-specific domain when a superfamily has several families. For example, for the zf-C2H2 superfamily, we classified proteins with both zf_C2H2 and ZBTB domains into the ZBTB family and proteins containing only zf_C2H2 domain into the zf_C2H2 family. The second rule is that if a TF has several different DBDs, it is assigned to the family with the smallest E value of DBD. The third rule is removing enzyme proteins since they were annotated as enzymes although they contain some DBDs.

### Optimized TF and cofactors prediction pipelines

**Figure 1.**
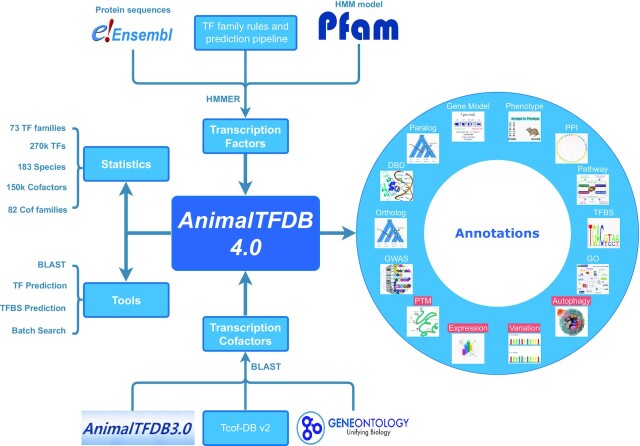
The workflow, data and annotation summaries of AnimalTFDB 4.0. The top of the figure illustrates the TFs prediction workflow, and the bottom of the figure describes the TcoFs prediction workflow. The left of the figure shows the data statistics and tools in AnimalTFDB 4.0. The right of the figure shows the multiple types of annotations in AnimalTFDB 4.0, of which variations, gene expression, PTMs and autophagy are newly added.

We built the TF prediction pipeline based on the TF family and classification rules. Firstly, we downloaded the Hidden Markov Model (HMM) profiles for DBDs of 58 TF families from the latest Pfam database (version 35.0) (23). Secondly, we reconstructed the remaining 14 TF families based on the DBD sequences from classical species (human, mouse, zebrafish, and fly) with HMMER (v3.1b2) (42). Thirdly, we used the hmmsearch program in the HMMER package to search all protein sequences of each species against the DBD HMM profiles to predict TFs (Figure 1). The E-value thresholds for each family were shown in Supplementary Table S2. Since the DBD HMM profiles and protein sequences were updated, we adjusted the E-value thresholds used in AnimalTFDB 3.0 for some families by manually checking human prediction results. For instance, the previous E-value threshold of Fork_head domain was 1e–4 and we adjusted it to 1e-3 because FOXO3B and FOXD3 can only be predicted using *E*-value 1e–3. Similarly, we adjusted the E-value threshold of Homeobox domain to 1e–2. Furthermore, we examined the ‘Others’ family and removed some proteins that are not TFs, such as centromere proteins (CENPA, CENPS, CENPT, CENPX).

As a result, in AnimalTFDB 4.0, we predicted a total of 1659 human TFs, which is consistent with 99.33% TFs in the previous version. The deleted TFs were SMARCA1, CCDC88A, ZBED5 and centromere protein genes, which have no relevant evidence to prove that they are TFs. The added TFs are NFILZ, FOXO3B, ZNF738 etc., all of which have DNA-binding TF activity in publications or GeneCards database annotation (43). We also compared the human TFs in AnimalTFDB 4.0 with those in The Human Transcription Factors Database (1) and found that among the 1639 TFs in The Human Transcription Factors Database, 1556 (94.93%) of them (1499 TFs and 57 TcoFs) are in our AnimalTFDB 4.0. The remaining 83 genes (5.06%) were commented on their website as ‘Likely to be sequence specific TF’ or without literature evidence. However, the majority of the unique 160 TFs in AnimalTFDB 4.0 were explicit TFs, including transcriptional activators (HSFX3, HSFX4, SMAD2, SMAD6, SMAD7, UBTF, TCF19, TCF25 etc.) and repressors (LRRFIP1, LRRFIP2, MIER1, MIER3, ID1/2/3/4 etc.). These comparisons provide good evidence for the high accuracy of our TF prediction results.

For TcoFs, we collected 1024 human TcoFs from AnimalTFDB 3.0, Tcof-DB v2 database (44) and GO database based on the related GO terms (‘transcription coactivator activity’, ‘transcription corepressor activity’, ‘transcription cofactor activity’, ‘regulation of transcription’, ‘chromatin remodeling’, ‘chromatin-mediated maintenance of transcription’, ‘histone *ylation’, ‘histone *ylase activity’ and ‘histone *transferase activity’). TcoFs in the other 182 species were identified by performing reciprocal best-hit BLAST between each of them and human with *E*-value ≤1e–4, identity ≥30% and coverage ≥50% (Figure 1).

## DATA SUMMARY AND OVERALL FUNCTIONS

In AnimalTFDB 4.0, we identified 274 633 TFs and 150 726 TcoFs in 183 animal species (Figure 1). The numbers of TFs and TcoFs for each species are shown in Supplementary Table S3, and there are 1659 TFs (8.30% in protein-coding genes) and 1024 TcoFs (5.12%) in human. The data demonstrate that the number of TF family ranged between 58 and 73, with most species (95.08%) having at least 70 TF families. Meanwhile, the number of TcoF families ranged from 56 to 82, and 181 species have at least 74 TcoF families. To provide comprehensive information for TFs and TcoFs, we collected various types of annotations, including ‘Ortholog’, ‘Paralog’, ‘DBD’, ‘GWAS’, ‘Gene Model’, ‘Gene Phenotype’, ‘PPI’, ‘Pathway’, ‘TFBS’ and ‘GO’. In AnimalTFDB 4.0, some new annotations were added, which include variations, gene expression, post-translational modifications and autophagy regulation information (Figure 1).

Users can browse AnimalTFDB 4.0 by species to obtain the list of TFs and TcoFs for individual species. Users can also browse by family to acquire the distribution of gene counts across species for a single family. Besides the data browsing function, we provided several functional tools in AnimalTFDB, including TF prediction, TFBS prediction, BLAST and batch search. The TF prediction function allows users to identify potential TFs in their uploaded protein sequences using the prediction pipelines in AnimalTFDB 4.0 (Figure 1). The TFBS prediction function can predict potential TFBS in user uploaded DNA sequences using the TFBS MEME files from HOCOMOCO (25), TRANSFAC (26), JASPAR (27), CIS-BP (28) and hTFtarget (14) (Figure 1). In BLAST function, TF protein sequences of all species or a specific species could be selected to perform BLAST search (Figure 1). For user's convenience, we added the batch search function allowing users to search AnimalTFDB 4.0 more flexibly (Figure 1).

## NEW ANNOTATIONS FOR TFS AND COFACTORS

### Variations

**Figure 2.**
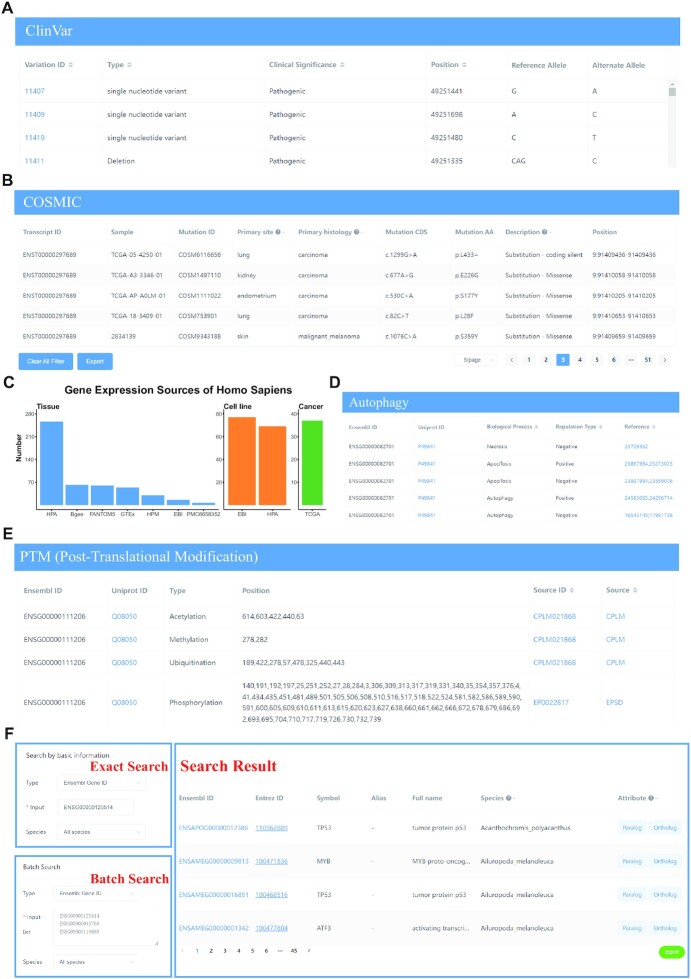
New features of AnimalTFDB 4.0. (**A**) The ClinVar variations of human TFs and TcoFs. (**B**) The COSMIC mutations of human TFs and TcoFs. (**C**) The gene expression sources of *Homo Sapiens*. The X-axis is the gene expression datasets and the Y-axis is the number of tissues, cell lines and cancer types. (**D**) The autophagy related information. (**E**) The post-translational modification information. (**F**) The exact and batch search function.

TFs act as essential regulators in the transcription process and their variations may change their protein sequences and functions, leading to transcriptional dysregulation. We collected the variation information from the latest ClinVar (16) and COSMIC (v96) (17) databases, which aggregated human health and disease related genomic variations. There are 898 (54.12%) human TFs and 706 (68.94%) TcoFs having a total of 190 627 variations in ClinVar, with an average of 118 records per gene. For each ClinVar variation record in TF or TcoF, the variation ID, variation type, position and clinical significance were shown in Figure 2A. In addition, almost all human TFs (98.25%) and TcoFs (99.21%) could find variants records in COSMIC, with a total of 8 294 851 COSMIC variants, all of which are somatic mutations in 119 human cancers. Among these data, about half mutations occurred in the coding sequence (CDS) and half in the 3’UTR, 5’UTR or intron regions. Mutations occurring in the CDS region were classified into 14 types, of which the major mutation types are missense (69.47%), coding silent (synonymous) (16.27%), and nonsense (6.36%), all of which belong to substitutions (Supplementary Table S4). Since there are so many mutations per gene, we displayed the related COSMIC information and provided a filtering function in AnimalTFDB 4.0 (Figure 2B). Users can filter the primary site, primary histology and description according to their needs. Moreover, users can clear all filters by clicking the clear button and export results by clicking the export button (Figure 2B).

### Gene expression

In AnimalTFDB 4.0, we provided gene expression information of TFs and TcoFs for 38 species, doubling the data volume from previous version. These expression data were from normal tissues, cell lines, different stages and cancers in human and other species. As a summary, expression data were available for 81.48–100% TFs and 89.34–100% TcoFs in 38 species (Supplementary Table S5). Expression data are classified into mRNA and protein expression, both of them are available for humans, while only mRNA expression data are available for other 37 species. Besides gene expression data in previous version, we collected more large-scale gene expression datasets for human, including gene expression in 54 non-diseased tissue sites from GTEx project (34), 60 tissues from FANTOM5 project (36), and The Human Protein Atlas (38) based on RNA-seq of gene expression in 256 tissues and 69 cell lines (Supplementary Table S6). In total, we collected gene expression data in different tissues, cell lines and cancers from eight datasets for human (Figure 2C). In addition, we collected large-scale gene expression data from some articles for more species, such as gene expression in seven organs across different developmental stages for 7 mammal species (39) and gene expression for nine species in different developmental stages and tissues (40) (Supplementary Table S6).

### Autophagy regulation information

Autophagy is a complex and vital process that controls cellular remodeling and quality control (45), and many TFs are involved in regulating autophagy (46). We gathered information about whether a TF or TcoF is involved in regulating autophagy-related processes from the THANATOS (31) database. Autophagy-related processes include autophagy, apoptosis, and necrosis. The regulation modes include positive regulation, negative regulation or both. In total, we collected 1,023 autophagy records from six mode organisms (*Homo sapiens*, *Mus musculus*, *Rattus norvegicus*, *Caenorhabditis elegans*, *Danio rerio* and *Drosophila melanogaster*) involving 338 TFs and 382 TcoFs. The autophagy regulation information for each TF or TcoF was shown in Figure 2D.

### Post-translational modifications

Post-translational modifications influence the functional regulation of TFs and their co-regulators (47). Growing evidence shows that the PTMs of TFs have positive and negative consequences on transcription (48). Here, we parsed 38 943 lysine modification sites (including 14 041 acetylation, 1169 methylation and 23 733 ubiquitination) from CPLM (29) database and 131 378 phosphorylation sites from EPSD (30) database in eight model species (*H. sapiens*, *M. musculus*, *R. norvegicus*, *C. elegans*, *B. taurus*, *Cavia porcellus*, *Gallus* and *D. melanogaster*). There are 2941 TFs and 2343 TcoFs with PTM information containing 1588 human TFs, 1013 human TcoFs, 980 mouse TFs and 835 mouse TcoFs, as well as 373 TFs and 494 TcoFs in the remaining six species (Supplementary Table S7). For each TF or TcoF, the positions of PTMs were shown in Figure 2E.

### Exact and batch search function

Besides the TF/TFBS prediction and BLAST functions on AnimalTFDB, for user's convenience, we added the exact search and batch search function allowing users to search the database flexibly. Users can search by entering the gene ID, transcript ID, protein ID or gene name or gene alias to find matched results in database (Figure 2F). Users can also make a batch search by inputting a list of genes (gene ID, transcript ID, protein ID and gene name are accepted) (Figure 2F). The batch search can guide downstream analysis in some situations. For example, after identifying the differentially expressed genes (DEGs) between two groups, users can put all DEGs as the input of ‘Batch search’ to determine which of them are TFs and explore their functions. By exact search or batch search, users can obtain gene ID, gene name, species and attributes (types of gene annotation information). Users can filter species and attributes and export results by clicking the export button (Figure 2F).

## SUMMARY AND FUTURE PERSPECTIVES

With the increasing sequenced and well annotated animal genomes, we updated AnimalTFDB to version 4.0 with new features. AnimalTFDB 4.0 provides 274 633 TFs and 150 726 TcoFs from 183 animal genomes. In addition, we added several new annotations including TF-related variations, gene expression, PTMs and autophagy information, as well as new functions such as the exact and batch search. Variation information of human TFs from ClinVar and COSMIC will provide useful resources for researchers to further explore the TF mutations and their associated diseases. More comprehensive TF expression information will help users better understand the relationship between TF and development as well as the diversity of TF expression. The PTM site and autophagy regulation information will be essential resources for studying the function and regulation of TF. The exact search and batch search functions will allow users to search AnimalTFDB flexibly. In summary, we believe these improvements will make AnimalTFDB more comprehensive and useful. Undoubtedly, the genomic data for various species will continue to grow. We will continue regularly updating the AnimalTFDB database to make it as a core resource for TF regulation.

## DATA AVAILABILITY

AnimalTFDB 4.0 is publicly accessible for worldwide users without any registration or login. Users can freely access all data in AnimalTFDB 4.0 at http://bioinfo.life.hust.edu.cn/AnimalTFDB4/.

## Supplementary Material

gkac907_Supplemental_File
